# How Search Engine Data Enhance the Understanding of Determinants of Suicide in India and Inform Prevention: Observational Study

**DOI:** 10.2196/10179

**Published:** 2019-01-04

**Authors:** Natalia Adler, Ciro Cattuto, Kyriaki Kalimeri, Daniela Paolotti, Michele Tizzoni, Stefaan Verhulst, Elad Yom-Tov, Andrew Young

**Affiliations:** 1 United Nations International Children's Emergency Fund (UNICEF) New York, NY United States; 2 ISI Foundation Torino Italy; 3 The Governance Lab New York University New York, NY United States; 4 Microsoft Research Herzeliya Israel

**Keywords:** internet data, India, suicide, mobile phone

## Abstract

**Background:**

India is home to 20% of the world’s suicide deaths. Although statistics regarding suicide in India are distressingly high, data and cultural issues likely contribute to a widespread underreporting of the problem. Social stigma and only recent decriminalization of suicide are among the factors hampering official agencies’ collection and reporting of suicide rates.

**Objective:**

As the product of a data collaborative, this paper leverages private-sector search engine data toward gaining a fuller, more accurate picture of the suicide issue among young people in India. By combining official statistics on suicide with data generated through search queries, this paper seeks to: add an additional layer of information to more accurately represent the magnitude of the problem, determine whether search query data can serve as an effective proxy for factors contributing to suicide that are not represented in traditional datasets, and consider how data collaboratives built on search query data could inform future suicide prevention efforts in India and beyond.

**Methods:**

We combined official statistics on demographic information with data generated through search queries from Bing to gain insight into suicide rates per state in India as reported by the National Crimes Record Bureau of India. We extracted English language queries on “suicide,” “depression,” “hanging,” “pesticide,” and “poison”. We also collected data on demographic information at the state level in India, including urbanization, growth rate, sex ratio, internet penetration, and population. We modeled the suicide rate per state as a function of the queries on each of the 5 topics considered as linear independent variables. A second model was built by integrating the demographic information as additional linear independent variables.

**Results:**

Results of the first model fit (*R*^2^) when modeling the suicide rates from the fraction of queries in each of the 5 topics, as well as the fraction of all suicide methods, show a correlation of about 0.5. This increases significantly with the removal of 3 outliers and improves slightly when 5 outliers are removed. Results for the second model fit using both query and demographic data show that for all categories, if no outliers are removed, demographic data can model suicide rates better than query data. However, when 3 outliers are removed, query data about pesticides or poisons improves the model over using demographic data.

**Conclusions:**

In this work, we used search data and demographics to model suicide rates. In this way, search data serve as a proxy for unmeasured (hidden) factors corresponding to suicide rates. Moreover, our procedure for outlier rejection serves to single out states where the suicide rates have substantially different correlations with demographic factors and query rates.

## Introduction

### Background

According to the World Health Organization (WHO), close to 800,000 people die by suicide every year, with 78% of global suicides occurring in low- and middle-income countries [1]. Teenagers and young adolescents are particularly at risk, as suicide represents the second leading cause of death among 15-29-year-olds worldwide [1]. These concerning figures do not even fully capture the magnitude of the problem. The WHO estimates that good quality data on suicide exist for only 60 countries worldwide.

According to official statistics, India is home to 20% of the world’s suicide deaths [2], yet the issue attracts limited national public health attention [3]. In addition, statistics on suicides released by the Indian National Crime Records Bureau (NCRB) are insufficient to understand the magnitude of the problem. In 2013, the NCRB reported that 134,799 people died of suicide, making the suicide rate 11% of total deaths [4]. However, evidence from other studies shows that the NCRB’s suicide rate data are grossly underreported. For instance, the WHO reported 170,000 cases of suicide deaths in India, which is about 35,000 higher than the NCRB’s data [3]. Similarly, the Registrar General of India implemented a nationally representative mortality survey indicating that about 3% of the surveyed deaths (2684 of 95,335) in individuals aged 15 years or older were due to suicide, corresponding to about 187,000 suicide deaths in India in 2010 (as reported in Patel et al [3]).

The factors contributing to these inconsistencies are likely manifold, including both data collection barriers and cultural challenges. The deep-rooted stigma associated with mental disorders, coupled with limited suicide prevention and mental health services, makes it difficult to address suicide as a major public health problem in India [5]. Until recently, suicide was a criminal offense in the country, likely compelling families to report suicides as death by an illness or accident so as to avoid punishment [6]. Moreover, analysis (if any) of suicide records is limited to demographic correlations. Patel et al [3], for instance, only focused on age and sex variables to analyze the survey findings.

Furthermore, there is little research on the important role played by stigma in suicide reporting in India, with the majority of the studies stressing the necessity of further research for a systematic assessment. Some existing studies, even if they do not provide a systematic assessment of the topic, report a connection between suicide reporting and stigma. Merriott [7], in a study of factors associated with the farmer suicide crisis in India, acknowledges the presence of stigma associated with suicide underreporting: “The NCRB figures, for which the studies in the introduction proposing an increasing farmer suicide rate come from, are considered significant underestimates as, for example, they only use police records to classify deaths, and due to the stigma associated with suicide in a country where it was illegal until a government decision in 2014.” Bhise and Behere [8] stress the presence of stigma related to mental illnesses and suicide prevention without proceeding to an assessment of stigma: “Creating a referral network of government and private hospitals and mental health professionals, training health professionals in identifying high-risk farmers, and strategies aimed at reducing stigma attached to mental illness will go a long way in suicide prevention.” Similarly, the study by Aggarwal [4]refers to the stigma related to suicidal behavior: “The anticipated changes as a result of this policy shift include: accurate reporting and recording of suicide as a cause of death, reduction in stigma associated with suicidal behavior and use of these figures to inform suicide prevention strategies.” A case study in Pakistan by Kahn et al [9] states that “the absence of systematic sampling of police data in societies with high social stigma will oversample people with severe mental illness, suggests selection bias and probably invalidates the results.” Kennedy et al [10] demonstrated higher levels of stigma and higher levels of suicide literacy in a study conducted in the Australian rural farming communities, suggesting how best practices can be adapted to improve stigma reduction and suicide prevention efforts.

Finally, a study by Armstrong and collaborators [11] focuses on how media reporting of suicide news in India performs against the WHO guidelines, stressing how strategies should be devised to boost the positive contribution that media can make to suicide reporting and prevention.

This paper seeks to contribute to this discussion by describing how data gaps in Indian suicide reporting can be filled through the creation of data collaboratives. Data collaboratives are “an emerging form of public-private partnership in which actors from different sectors exchange information to create new public value.” [12]. Data collaboratives are increasingly being tested as a means for improving evidence-based policy making and targeted service delivery around the world, including, notably, data-sharing arrangements between corporations and national statistical offices [13].

The main goal of this paper is to shed some light on the public health issues of suicides among the Indian population through the lens of Web data. Can data about Web-based information-seeking behavior help to study the determinants for suicides in the various Indian states? In particular, by combining official statistics on suicide with demographic information about the population and data generated through search queries on various keywords related to suicide, this paper seeks to: add an additional layer of information to more accurately represent the magnitude of the problem, determine whether search query data can serve as an effective proxy for studying factors contributing to suicide that are not represented in traditional datasets (eg, search queries for specific keywords related to means of suicide or to social factors that can influence the mental status of the person, such as economic difficulties or academic pressure for young people), and consider how data collaboratives built on search query data could inform future suicide prevention efforts in India and beyond.

### What Is Known About Those Who Die by Suicide in India

The literature varies and sometimes offers a convoluted picture of who is at risk of dying by suicide, both in India and beyond. There seems to be a consensus that young people aged 15-29 years are particularly at risk. Patel et al [3] found that 40% of suicides among men and 56% of suicides among women occurred between the ages of 15 and 29 years. However, while the prevalence of suicide among the young is generally accepted, the male-to-female suicide ratio in India varies greatly, ranging from 1.04 to 1.63 according to some studies [6], while official statistics from the NCRB show a male-to-female suicide ratio of 2:1. Patel et al [3], on the other hand, found an age-standardized rate per 100,000 people aged 15 years or older of 26.3 for men and 17.5 for women, demonstrating the need for more clarity. The paper also found that Indian boys and men had a 1.7% cumulative risk of dying by suicide between the ages of 15 and 80 years compared with a 1.0% risk among girls and women [4].

There exists a strong correlation between educational backgrounds and suicide risks, with the less educated accounting for 70.4% of suicide cases as recorded in the NCRB data [6]. Counterintuitively, suicide among students in India is also increasing, moving from 5.5% of total suicides in 2010 to 6.2% in 2013 [14].

Suicide rates vary by occupation [4]. Housewives accounted for about 18% of the total victims, while farmers comprised 11.9% of the total victims followed by those working in the private sector (7.8%), unemployed (7.5%), and those working in the public sector (7.8% and 2.2%, respectively). A study reported that approximately 16,000 farmers in India die by suicide every year [7]. Patel et al [3] found that about half of suicide deaths in India arose from poisoning, especially resulting from the ingestion of pesticides.

### Risk Factors

Some population groups are more at risk than others. For example, a study shows that 27.2% of primary care patients suffer from depressive disorder, and 21.3% of them have attempted suicide, demonstrating how depression is 1 of the underlying factors that drives suicide [15].

Risk factors cut across geographical lines, with official statistics showing a significantly higher rate of suicide taking place in southern states, such as Tamil Nadu, Andhra Pradesh, Karnataka, Kerala, West Bengal, and Maharashtra, where 63.6% of suicide cases occurred [4]. South India is the area encompassing the Indian states of Andhra Pradesh, Karnataka, Kerala, Tamil Nadu, and Telangana as well as the union territories of Lakshadweep, Andaman and Nicobar Islands, and Puducherry, occupying 19% of India’s area and with about 18% of the total population of India.

These data paint a picture of the breadth and diversity of the suicide issue in India. But given the stigma associated with suicide, poor quality data, and the still-recent decriminalization of suicide attempts, these statistics confuse as much as they elucidate. The country’s underreporting challenge—and the likely neglect of certain population groups altogether—creates major challenges for meaningfully determining who is at risk.

### Internet Data as a Source for Health Information

In response to the issue of suicide underreporting in India, this paper looks at a specific cohort of people *(* English-speaking internet users*)* to add another layer of understanding about those at risk of suicide. The majority (one-third) of internet users are young (18-35 years) [16], which coincides with the age group most at risk for suicides (15-29 years).

With its 360 million users (26% of the population), India is home to more internet users than any country save China [17]. Men dominate internet usage in India [18], with 71% to women’s 29%. Internet usage is more prevalent in northern (27%) and western states (25%) compared with the South (19%) and East (16%) [16]. Yet, not all Indians accessing the internet do so in English. The Indian Constitution recognizes 22 official languages, and the number of Indian-language internet users has grown dramatically over the years, surpassing English users: 234 million compared with 175 million, respectively.

Internet data have been used to monitor health behaviors for a variety of conditions ranging from infectious diseases [19-21] to mental health conditions [22]. Social media is one source of internet data that has provided several insights on suicides [23-25]. However, as opposed to social media, anonymous Web-based venues, especially search engines [26], allow people to seek information on sensitive topics. Monitoring such venues can, thus, offer a window into behaviors that are otherwise difficult to study. Specifically, in the case of suicides, social media has been used to detect suicidality [27], and search engine logs were utilized to analyze suicides in general [28-30] and the Werther Effect (copycat suicides) in particular [21]. Additionally, search volume for past versus future was shown to be a predictor of suicide rates in the United States [31].

Here we examine internet search engine logs for information about suicides. Search engine logs, as analyzed here, focus on a population of English-speaking people in India. The market share of Bing in India was reported to be around 7% at the time of data collection [32]. Moreover, as shown in Fisher and Yom-Tov [33], people seeking information on suicides via search engines are (at least in the United States) people who are contemplating suicide, not people who may necessarily die by suicide.

### Internet Search Queries as a Source for Suicide-Related Information

This paper’s methodology, described below, builds on previous work leveraging search query data analysis. Numerous studies have found that search query data are reflective of behaviors in the physical world [34]. In the United States, for example, people searching for actionable information about suicides (how to kill themselves) correspond to the population that attempts suicide—but not the population that successfully suicides [33].

Several studies have analyzed Google Trends, an aggregate measure of search query volume, and found correlations between search queries for suicide and the rate of suicide. Gunn and Lester [28] found a correlation between the volume of queries about suicide and the actual number of suicides by analyzing search words and phrases like “how to suicide.” Hagihara et al [29] conducted a study in Japan that shows how suicide queries spike in the period before there is an increase in suicide rate [24]. This method was replicated in Taiwan and Australia, but those studies yielded contradicting results [28]. Other studies are most skeptical about the correlation between Google queries and suicide rates, concluding that a tool to identify relevant search queries must be further developed to create a more precise modeling mechanism [35].

Finally, Kristoufek et al [36] studied how data on the number of Google searches for the terms “depression” and “suicide” in England related to the number of suicides between 2004 and 2013. The researchers found that estimates drawing on Google data were significantly more accurate than estimates relying on previous suicide data alone. Interestingly, their findings show that a greater number of searches for the term “depression” is related to fewer suicides, whereas a greater number of searches for the term “suicide” is related to more suicides, though the correlation is not extremely high (*R*^2^ of about 0.4).

## Methods

### Search Engine Data

We extracted all English language queries from the Bing search engine submitted by people from India between November 2016 and February 2017 (inclusive). For each query, we recorded the time and date of the query, the state in India from where the user made the query, and the text of the query. The correlation between the number of queries per state and the population of that state multiplied by internet penetration provided a positive Spearman correlation (ρ=0.93, *P*<.001).

The queries on 5 topics were identified by testing whether the text of the queries contained 1 or more of the inclusion terms in Table 1 and did not contain any of the exclusion terms. The exclusion terms were found by identifying the most common words and word pairs appearing in conjunction with the inclusion terms and identifying those that were unrelated to suicidal intentions.

### State Data on Suicide Rates

The suicide rate per state was obtained from the latest available data, the 2014 Accidental Deaths & Suicides in India report from the NCRB [37] (see Multimedia Appendix 1).

### Demographic Data

In the data analysis, we have included demographic information at the state level, including urbanization, growth rate, sex ratio, internet penetration, and population. Data were obtained as follows:

1. Sex ratio, population, urbanization, and growth rate: from Wikipedia [38].
2. Income: per capita national income 2013-2014, available from the India National Informatics Centre [39].
3. Internet penetration: from data published by The Hindu newspaper [40].
4. Enrollment in higher education: gross enrollment ratio in higher education, available from the Statistical Year Book of India, 2016 [41].

### Statistical Modeling of Search Engine Data

Data were analyzed for their temporal patterns (diurnal and weekly) as well as their variation by state. We modeled the reported suicide rate per state as a function of the fraction of queries on each of the 5 topics from each state. Thus, the dependent variable in our models was the expected number of suicides in each state, which is the product of the reported suicide rate multiplied by the size of the population. The independent variables included the fraction of queries (with respect to the number of internet users in the state) from each state for each topic. Outliers were removed using an iterative process: up to either 3 or 5 states were removed by finding the state which, if removed, increased model fit (*R*^2^) by the greatest amount and repeating this process 3 or 5 times, as desired. The model used throughout is a linear model, unless otherwise stated. We report model fit for different levels of outlier rejection below.

### Risks and Data Responsibility

To be clear, the use of data related to suicide, suicidal ideation, and mental health creates some level of risk across the data lifecycle. The analysis described in this paper adhered to strict data responsibility principles, ensuring that sensitive data were not shared or compromised and that aggregated rather than personally identifiable data informed our findings.

For the field at large, to effectively and legitimately leverage data collaboratives to improve public understanding of suicide rates and devise evidence-based prevention strategies, data responsibility methods and tools are needed for both sides of data-sharing arrangements.

**Table 1:** Exclusion and inclusion terms for each of the 5 topics related to suicides.

Topic	Inclusion terms	Exclusion terms
Suicide	“suicide,” “kill myself”	“suicide squad,” “song,” “download,” “skill,” “killer,” “movie,” “video,” “bill,” “game,” “lyrics,” “mp3,” “suicide girl,” “militia,” “mockingbird,” “ghandi,” “akame ga kill,” “3 days to kill,” “wifi kill,” “kill dil,” “kill zone,” “killzone,” “kill em with kindness,” “kill me heal me,” “rkill”
Depression	“depression,” “depressed”	—^a^
Hanging	“hang,” “hanging”	“wall hanging,” “hanging garden,” “macrame”
Pesticide	“pesticide”	—
Poison	“poison”	“poison ivy,” “poisonous snakes,” “poisoned thoughts,” “poison thoughts,” “food poisoning,” “hanging boobs,” “hanging lights”

^a^No exclusion terms considered.

The research that informed this paper is contributing to the development of data responsibility frameworks to aid the field in assessing if, when, and how data can be shared in a responsible manner as part of a data collaborative. This study is considered exempt by the Microsoft Institutional Review Board.

## Results

### Temporal Analysis

**Figure 1:**
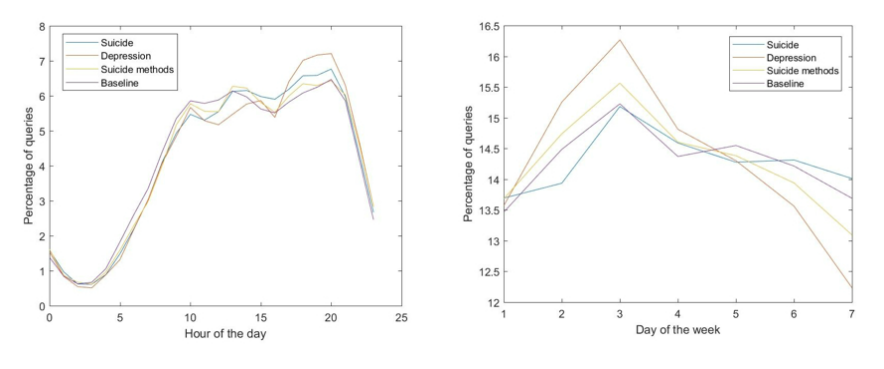
Diurnal and weekly patterns of relevant queries (suicide, depression, and suicide methods) compared to the baseline of all queries made in India.

Figure 1 shows the percentage of queries about suicide, depression, and suicide methods as a function of the hour of the day and the day of the week. Days are numbered sequentially from Sunday (1) through Saturday (7). As these figures show, these queries broadly follow the baseline (all queries made in India). However, closer inspection reveals that relevant queries are approximately 20% less likely, compared with the baseline, during early morning hours and up to 15% more likely during the evening to late night hours. The largest difference between the baseline and relevant queries when stratified by day of the week is smaller than 5%.

### Using Query Data to Model Suicide Rate

Table 2 shows the model fit (*R*^2^) when determining the suicide rates from the fraction of queries in each of the 5 topics as well as the fraction of all suicide methods. States with <0.25% of the Indian population are excluded (n=20). As the table shows, the correlation increases significantly with the removal of even 3 outliers and improves slightly when 5 outliers are removed. In all cases, a statistically significant correlation is reached, but the best correlation is obtained for suicide methods (hanging, pesticide, and poison) and only to a lesser extent for depression. This indicates that people who are considering suicide are not only just asking about the term itself but also about possible precursors (depression) and methods of suicide.

We next analyzed the outliers and whether, given a model that was constructed after the exclusion of an outlier, more suicides would be modeled by query volume compared with official statistics, or vice versa. A negative outlier would, thus, indicate that more suicides are determined by the model according to the volume of queries in a topic than are reported by official data. A positive outlier would indicate the reverse: the official reported suicide rate is greater than that which would be inferred from the queries.

Analyzing the models after excluding 5 outliers per model, we find that there are slightly more negative outliers than positive ones: 16 negative outliers compared with 14 positive outliers.

**Table 2:** Model fit for modeling the expected number of suicides in each state from the fraction of queries in each topic.

Query	*R*^2a^ (no outliers removed)	*R*^2^ after removal of 3 outliers	*R*^2^ after removal of 5 outliers
Hanging	0.29	0.49	0.65
Pesticide	0.16	0.71	0.80
Poison	0.33	0.65	0.72
All methods of suicide (hanging, pesticide, and poison)	0.47	0.68	0.80
Suicide	0.13	0.65	0.79
Depression	−0.01	0.34	0.50

^a^*R*^2^: model fit.

**Figure 2:**
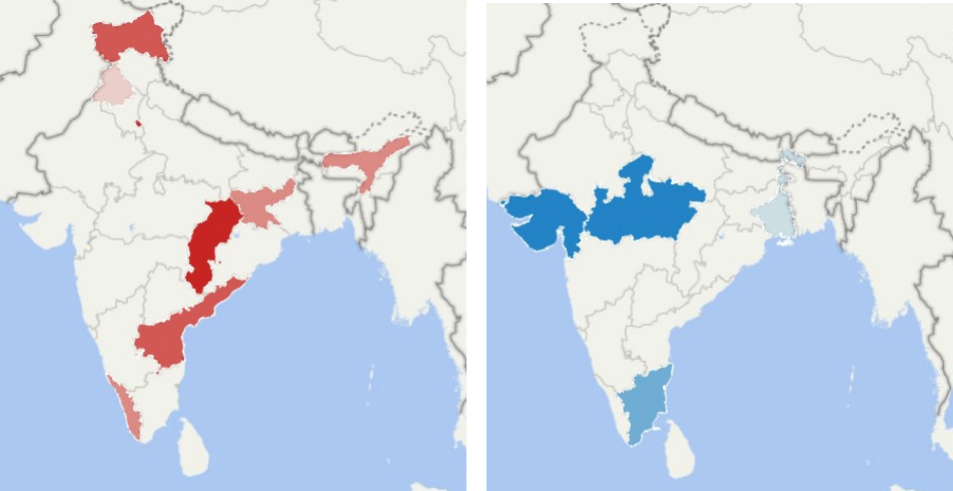
Maps showing states that are negative outliers (more suicides modeled by Web data than reported), left, and positive outliers (fewer suicides modeled by Web data than reported), right. Darker colors indicate that the state is an outlier in more terms.

Figure 2 shows the outliers. From this figure, it can be seen that the outliers are not distributed randomly; in all but 1 case, a state will either have all positive or all negative outliers. The states with most negative outliers are Jammu & Kashmir and Jharkhand (4 and 3 outliers, respectively), and the ones with most positive outliers are Telangana and Gujarat (5 and 4 outliers, respectively).

Our findings singled out 2 states, Jammu & Kashmir and Jharkhand, as having more queries indicative of suicide than would be expected, given the published suicide rates in these states. In contrast, Gujarat and Telangana have more reported suicides than modeled by search data.

### Addition of Demographic Data to Query Data

Demographic data are correlated with suicide rate. Therefore, in this section, we investigate whether query data can add to the modeling of suicide rates, beyond what demographic data can provide. To overcome the dimensionality of additional variables, we employ a stepwise linear model, which selects the most significant variables under the criteria that the *P* value for an *F* test of the change in the sum of squared error is maximally reduced by adding a variable.

**Table 3:** Model fit for the expected number of suicides in each state from the fraction of queries in each topic, with and without demographics, using a stepwise model.

Query	Without demographic data		With demographic data	
	*R*^2^^a^ (no outliers removed)	*R*^2^ after removal of 3 outliers	*R*^2^ (no outliers removed)	*R*^2^ after removal of 3 outliers
Hanging	0.28	0.49	0.51^b^	0.75^b^
Pesticide	0.16	0.71	0.51^b^	0.91
Poison	0.33	0.65	0.51	0.76
All methods of suicide (hanging, pesticide, and poison)	0.47	0.68	0.47	0.74
Suicide	0.13	0.34	0.51^b^	0.75^b^
Depression	−0.01	0.65	0.51^b^	0.75^b^

^a^*R*^2^: model fit.

^b^Cases where query data were not selected for inclusion in the model.

**Table 4:** Outliers (with the rejection of 3 states; the direction of outliers is in parentheses) for pesticides and poison queries for models that use demographic data and query data and for models that only use query data. + and – indicate positive and negative directions, respectively.

Query		Demographics + query data model	Query data only
**Pesticides**			
	Andhra Pradesh	–	–
	Kerala	–	N/A^a^
	Punjab	–	–
	Jammu and Kashmir	N/A	–
**Poison**			
	Telangana	+	+
	Delhi	+	N/A
	Jharkhand	­–	–
	Madhya Pradesh	N/A	+

^a^N/A: not applicable.

Table 3 shows the model fit for models using solely query data and for models using both query data and demographic data. States with less than 0.25% of the Indian population are excluded (n=20)The variables selected when using just demographic data are urbanization rate and sex ratio, both positively correlated with suicide rates. Table 3 shows that for all categories, if no outliers are removed, demographic data can model suicide rates better than query data. However, when 3 outliers are removed, query data about pesticides or poisons becomes significant (fourth column) and improves the model over using only demographic data.

Using both demographics and query data, the outlying states for pesticides are all negative (more suicides modeled by Web data than reported), namely, Andhra Pradesh, Kerala, and Punjab. The outliers for poison are Telangana (positive), Delhi (negative), and Jharkhand (negative). Thus, the sole positive outlier is the same as using query data alone.

Comparing the outliers we obtained with the query data only with those obtained when including the demographics data, results are rather similar (Table 4). We report only outliers for pesticides and poison because these are the only keywords for which query data were selected for inclusion in the model.

## Discussion

Internet search data have been shown in previous work to serve as a proxy for many health-related behaviors, enabling the measurement of rates of different conditions ranging from influenza to suicide. Here we use these data to model suicide rates in India. Internet data can be influenced and biased by the population using it. Similarly, official suicide statistics might be susceptible to underreporting by statistical agencies. In our work, we, therefore, applied 2 tools that could mitigate these biases. First, we used both search data and demographics to enhance the understanding of official suicide rates. In this way, search data serve as proxy for unmeasured (hidden) factors corresponding to suicide rates. Second, our procedure for outlier rejection serves to single out states where the suicide rates have substantially different correlations with both demographic factors and query rates. To emphasize the difference between these 2 influences, consider the following simplifying cases as clarifying examples: First, suppose that in 1 state there exists a population where people kill themselves by suicide but does not use the internet to search for it beforehand. In that case, such a state would be identified as an outlier in our data. Similar cases would occur if there is underreporting in the state or some demographic factor not included in the model that does not influence the search queries. In this case, the state would be highlighted as a positive outlier. On the other hand, if a demographic factor that is unavailable to the model can help understand suicide rates and search queries are influenced by this factor, they will serve as a proxy to suicide rates and will improve model correlations. We believe that in the data analyzed, a mix of these effects is at work. Specifically, some (mostly agricultural states) were found to be negative outliers; in these states, it might be that a population who does not use the internet or the Bing search engine are those among which more suicides are reported. Similarly, several states were identified as positive outliers, suggesting that in those states, underreporting might be occurring, or there might be some other social or demographic factor at play that is not captured by the model and by the search queries activity. We do not know exactly which of these effects are at play. Thus, further investigation will be needed to disentangle social factors from actual suicide underreporting.

Cases in point are Telangana and Andhra Pradesh. The former was recently separated from the latter and declared an independent state. Both are states where agriculture is a major industry and, thus, where farmer suicides may be expected. However, the first of these was identified as a positive outlier (where fewer suicides were modeled based on query data) whereas the latter was identified as the opposite, for both models. Several explanations may be offered for this difference. First, Telangana has registered more suicides due to poverty and unemployment [37]. Second, Telangana has a higher urbanization rate compared with Andhra Pradesh (39% and 30%, respectively). Third, Telangana has a higher education rate (35% vs 29%). Both variables are taken into account by the demographics and could, thus, show a higher suicide rate in Telangana than is expected solely from query data. This is coupled with the fact that Telangana has recorded a recent increase in student suicides [42]. Finally, in an attempt to curb farmer suicides, Digital India has improved access to information by providing farmers with internet access [43], a factor which may have contributed to a higher than expected rate of queries from farmers in our data.

Regarding methods of suicide, queries for 2 methods were found to improve the modeling of suicide rate over demographics. Interestingly, searches for depression or the phrase “suicide” did not. This result has an important implication: efforts to model the incidence of suicide toward taking preventative action are more likely to find success if they focus on queries about specific methods of suicide as opposed to keywords related to depressive symptoms or the concept of suicide more generally.

Most internet search engines nowadays provide information on helpline numbers in highlighted information boxes above search results when users search for information on suicides. Such pointers to crisis support, particularly in smartphone apps, have been shown to be effective in some studies [44]. Our data on the diurnal variations and weekly variations of queries can help guide the staffing of suicide prevention helplines. Moreover, to the best of our knowledge, these boxes are only displayed when people explicitly search for the term “suicide.” Our results suggest that future research should investigate the display of these boxes also in cases where people search for methods of suicide. However, it is unclear how to distinguish between searches of methods that are related to suicides and those that are not (eg, a farmer searching for information on pesticides).

Public awareness campaigns may be a driver of people searching for information about suicides. However, in the data analyzed, we found that searches for methods of suicide are better correlated with the suicide rate. We suggest that this shows that our data are not affected by such exogenous factors. We also suggest that additional research is needed to explore the feasibility of creating new recommendations on the sale of pesticides in India, particularly for sales to young people.

Building on this work and drawing upon cross-sector data, including but not limited to search queries, we are conducting research aimed at increasing our understanding of the drivers of suicide among young people in India, how those drivers differ across regions, and how those findings can inform suicide prevention efforts in the country going forward.

## Appendix

Multimedia Appendix 1 Supplementary information on the data sources.

## Abbreviations

NCRB: National Crime Records Bureau
WHO: World Health Organization
