# Supplementary material for: How Search Engine Data Enhance the Understanding of Determinants of Suicide in India and Inform Prevention: Observational Study
Source: J Med Internet Res. 2019 Jan 4;21(1):e10179. doi: 10.2196/10179 (PMC6682304; doi:10.2196/10179)
Supplement: Multimedia Appendix 1 [file jmir_v20i12e10179_app1.pdf]

Multimedia Appendix 1. Beyond demographics: How search engine data can enhance the prediction of suicide rates in India

Demographic information were taken from:

Sex Ratio, Population, Urbanization, Growth Rate: http://www.census2011.co.in/states.php

Income: http://pib.nic.in/newsite/PrintRelease.aspx?relid=123563, 2013-2014

Internet penetration: www.thehindu.com/sci-tech/technology/internet/The-India-wide-web/article14588938.ece

Enrolment in higher education: http://www.mospi.gov.in/statistical-year-book-india/2016/198

Suicide Rates: http://ncrb.nic.in/StatPublications/ADSI/ADSI2014/chapter-2%20suicides.pdf

	Urbanization (%)	Total % enrolment in higher education	Sex ratio	Decadal Growth (%)	Density (inhabitants/km^2)	Internet penetration (%)	Population (% of tot)	Suicide rate (per 100,000)
**Andhra Pradesh**	29.58	29.2	0.993	11.1	303	28.11	4.08	12
**Assam**	14.08	15.4	0.958	16.9	397	20.79	2.58	11.1
**Bihar**	11.3	13.1	0.918	25.1	1102	14.25	8.58	0.7
**Chandigarh**	97.25	54.1	0.818	17.1	952	43	0.09	6.3
**Chhattisgarh**	23.24	13.9	0.991	22.6	189	19.65	2.11	22.4
**Delhi**	97.5	42.4	0.868	21.0	11297	95.51	1.38	10.3
**Goa**	62.17	25.3	0.973	8.2	394		0.12	15.1
**Gujarat**	42.58	19.4	0.919	19.2	308	33.16	5	11.7
**Haryana**	24.25	27.7	0.879	19.9	573	27.47	2.09	11.9
**Himachal Pradesh**	10.04	28.2	0.972	12.8	123	42.76	0.57	9.2
**Jammu And Kashmir State**	27.21	25.1	0.889	23.7	124	29.14	1.04	2.1
**Jharkhand**	24.05	12.4	0.949	22.3	414	14.25	2.72	4
**Karnataka**	38.57	26.2	0.973	15.7	319	36.25	5.05	17.8
**Kerala**	47.72	25.1	1.084	4.9	859	40.79	2.76	23.9
**Madhya Pradesh**	27.63	20.4	0.931	20.3	236	19.65	6	11.9
**Maharashtra**	45.23	25.2	0.929	16.0	365	36.94	9.28	13.9
**Manipur**	20.21	36.8	0.985	18.7	122	29.11	0.22	2
**Meghalaya**	20.08	19.7	0.989	27.8	132	29.11	0.24	3.6
**Mizoram**	51.51	22.1	0.976	22.8	52	29.11	0.09	11.1
**Nagaland**	28.97	14.9	0.931	-0.5	119	29.11	0.16	0.6
**Puducherry**	68.31	46.3	1.037	27.7	2598		0.1	40.4
**Punjab**	37.49	24.3	0.895	13.7	550	43.54	2.3	3.3
**Rajasthan**	24.89	19.1	0.928	21.4	201	23.63	5.67	6.3
**Tamil Nadu**	48.45	42.4	0.996	15.6	555	39.4	5.96	23.4
**Telangana**	38.66	35.1	0.993	17.8	307	28.11	2.97	26.5
**Uttar Pradesh**	22.28	21.1	0.963	20.1	828	17.03	16.49	1.7
**West Bengal**	31.89	16.2	0.950	13.9	1029	23.89	7.55	15.5
